# Identification of lipid regulatory genes modulated by polyherbal formulation in chicken liver tissues using transcriptome analysis

**DOI:** 10.5455/javar.2022.i611

**Published:** 2022-09-30

**Authors:** Saravanakumar Marimuthu, Subramaniyam Suresh, Prashanth D’Souza

**Affiliations:** R & D Centre, Natural Remedies Private Limited, Bengaluru, India

**Keywords:** CCL, oxidation, KP, lipogenesis, lipid metabolism, transcriptome

## Abstract

**Objective:**

To elucidate the cellular mechanisms of polyherbal formulation [Kolin Plus^TM^ (KP)], genomics was performed to delineate the genes and pathways associated with lipid regulation through transcriptional profiling of the liver in commercial broilers raised on diets deficient in choline chloride (CCL).

**Materials and Methods:**

The gene expression patterns were studied for four groups [normal diet: normal, choline chloride deficient (CCD), KP (400 gm/ton), and CCL (400 gm/ton)] using Agilent microarray on day 42. The hierarchical cluster analysis was carried out on 12,614 differentially expressed genes (DEGs) with a similar expression.

**Results:**

Out of 12,614 significant DEGs, 1,926, 448, and 1,330 genes were expressed at higher rates, and 413, 482, and 1,364 were expressed at lower rates than CCD (CCD *vs.* normal), CCL (CCL *vs.* CCD), and KP (KP *vs.* CCD), respectively. GO enrichment analysis of DEG further revealed the significant association of biological process items with the lipid, sterol, and lipoprotein metabolic processes. In particular, peroxisome proliferator-activated receptor gamma coactivator 1 alpha, carnitine palmitoyl transferase I, hydroxyacyl-CoA dehydrogenase trifunctional multienzyme complex subunit beta, and patatin-like phospholipase domain containing 2 genes involved in fatty acid oxidation and lipase C, ABCG5, ABCG8, acetyl-CoA carboxylase, ATP citrate lyase enzyme, and peroxisome proliferator-activated receptor gamma genes involved in lipogenesis were altered by KP intervention for lipid metabolism.

**Conclusions:**

These findings reveal that the supplementation of KP prevents fatty liver-associated problems in broiler chickens by modulating the expression of the above-mentioned genes that are responsible for the oxidation of fatty acids and lipogenesis in the liver.

## Introduction

Choline is a water-soluble vitamin, which is available as phosphatidylcholine or sphingomyelin in various foods [1,2], such as egg yolk, dried soybean, pork, wheat germ, and glandular meat [3]. It has three important functions in the body, namely maintaining the cell membrane integrity [4] and transmembrane signaling [5], being involved in the synthesis of neurotransmitters such as acetylcholine [6,7], and acting as the methyl donor and organic osmolyte after being oxidized into betaine [8]. In addition, choline has a critical function in lipid regulation by inhibiting abnormal fat accumulation in the healthy liver and gall bladder [9]. Phosphatidylcholine, a class of phospholipid with choline as a head group, is essential for forming very low-density lipoprotein, which mobilizes the hepatic tissue fat to the non-hepatic tissues [10]. Thus, choline deficiency would lead to a fatty liver [11,12], but with low body fat [13] in all animal species.

Unlike other vitamins, choline can be synthesized by all animal species through *de novo* synthesis when the diet contains a sufficient amount of methyl donor groups, such as methionine or betaine. However, chicks (below the age of 8 weeks) are not capable of synthesizing enough quantity, which leads to choline deficiency that results in growth retardation and perosis [14,15]. This was supported by several authors who reported that choline supplementation improved the growth performance and breast yield in commercial broilers [15–18]. So, choline was considered an important feed additive for chickens to help them grow and keep them away from getting perosis.

The disadvantages of synthetic choline chloride (CCL) made the researchers look for an herbal choline replacer. We have already demonstrated the choline-like effect of Kolin Plus^TM^ (KP) [19]. The only difference between a normal diet and a choline chloride deficient (CCD) diet in the above study was the amount of available choline, which elicited the negative effects of choline deficiency such as worsening of performance traits, changes in liver-marker enzymes, and liver histology indicating fatty liver [19]. Attempts were made to uncover the pathways and networks underlying fat metabolism using microarray technology, a promising tool in nutrigenomics that permits genome-wide differential gene expression analysis. Therefore, the current trial was planned to identify the molecular mechanisms that underlie the choline-like effect of KP through transcriptional profiling of broiler liver tissues.

## Materials and Methods

### Ethical approval

The experiment was carried out under ethical approval from the Institutional Ethics Committee vide No.: AHS/PR/01/2016, and all methods were carried out in accordance with the guidelines for animal experimentation.

### Kolin Plus^TM^

KP is a trademarked polyherbal formulation made by M/s. Natural Remedies Pvt. Ltd., Bengaluru, India, containing *Acacia nilotica* and *Curcuma longa*.

### Study design and experimental procedure

The study design and procedures for raising the chicken were published earlier [19]. On day 39, after weighing, six birds were randomly selected from four groups [normal diet: normal, CCD, KP (400 gm/ton), and CCL (400 gm/ton)] and sacrificed by jugular vein exsanguination and used for sampling of the liver tissues. Approximately 0.5 gm of liver tissues were collected and washed with precooled RNase-free phosphate-buffered saline, cut into pieces, and placed immediately in RNAlater® (Sigma-Aldrich, CA) for 12 h at 4°C and then stored at −80°C until further processing.

### Isolation, probe preparation, and hybridization

The RNA isolation, complementary RNA preparation and probe labeling, and microarray hybridization were carried out using the method described by D’Souza et al. [20].

### Microarray analysis

The microarray raw data collection and analysis were carried out as explained in our previous work, and the principal component analysis (PCA) was performed to check the data quality and to understand the gene expression variation across different groups [20].

### Identification and hierarchical clustering of differentially expressed genes (DEGs)

The fold change (logbase2) values for each gene were averaged for the replicate samples. The log-transformed normalized data was further interrogated for the DEGs. For each dataset comparison, the DEGs were selected with a cut-off criterion ±0.6 (fold change calculated as logbase2) and a *p*-value less than 0.05. Student’s *t*-test generates the *p*-value by looking at the similarity of the differential fold change across the replicate samples of the test against the control sample in replicates. This was carried out according to the procedure explained by D’Souza et al. [20].

### Bioinformatics

The bioinformatics evaluation was conducted as detailed in our previous work by D’Souza et al. [20], utilizing a public database and DAVID 6.7 online tools.

## Results and Discussion

### Performance parameters

The detailed trial report was published earlier [19]. In brief, the performance parameters, namely bodyweight and feed conversion ratio, were affected adversely in the CCD group compared to the normal group. However, administration of CCL and KP at 400 gm/ton reversed the negative impact on performance parameters, with a better response observed in KP.

### Detection of DEGs

**Figure 1.**
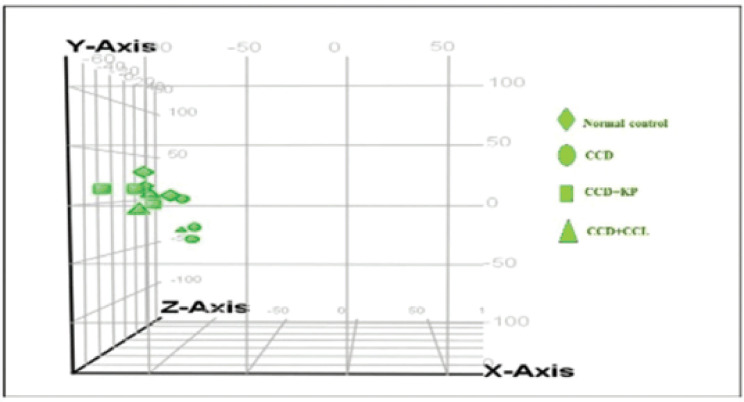
PCA scatter plot.

The Genespring GX was used to validate the intra-array normalized raw data. The PCA scatter plot is shown in Figure 1 and allows the viewer to view the separations between the groups of replicates. In the current study, replicates of normal, KP, and CCL groups were clustered together and separated from the arrays in the CCD group. Furthermore, the genes were compared between the groups, and 12,614 significant liver DEGs (cut-off criterion ±0.6-fold change calculated as logbase2) were identified using hierarchical clustering analysis. Out of which, 1,926, 448, and 1,330 genes were expressed at higher rates, and 413, 482, and 1,364 were expressed at lower rates in CCD (CCD *vs.* normal), CCL (CCL *vs.* CCD), and KP (KP *vs.* CCD), respectively.

### Biological function clustering

**Figure 2.**
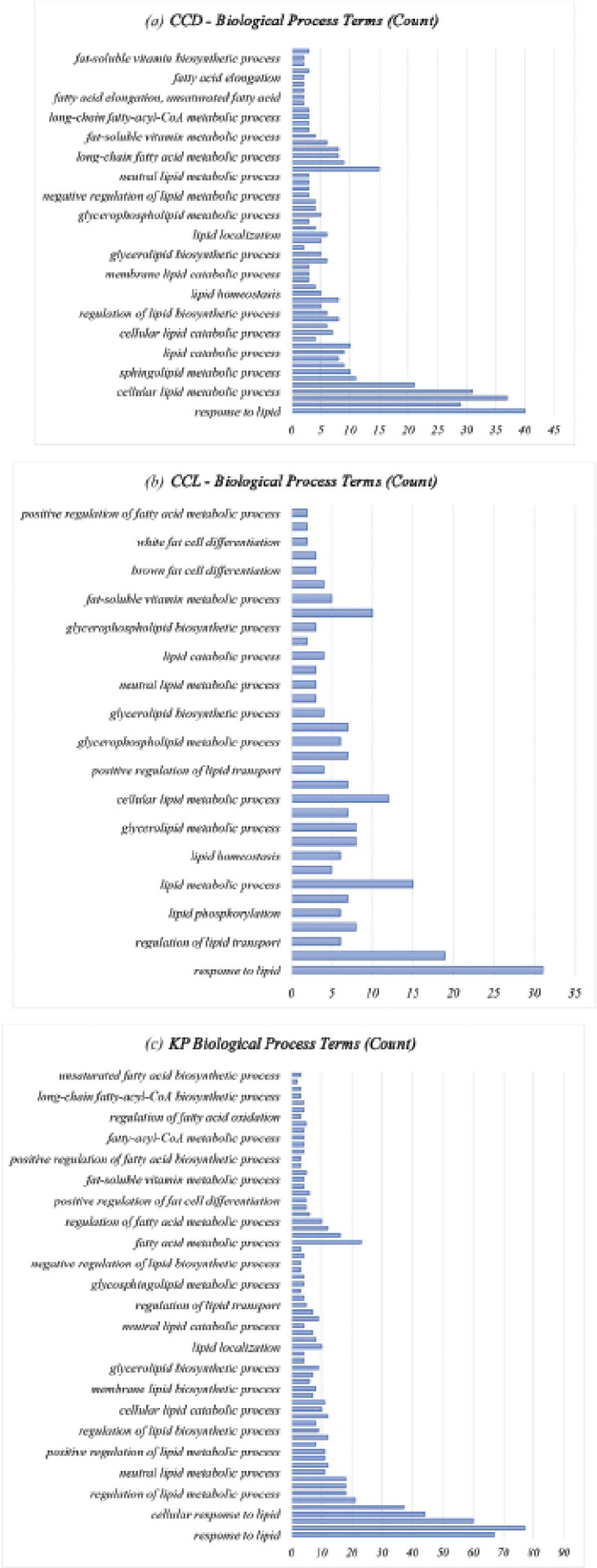
The biological process enriched items associated with (a) CCD, (b) CCL, and (c) KP.

The GO annotation was carried out to identify the key GO terms [biological processes (BP)] of genes significantly modulated by KP intervention. Because BP was the most relevant to the current results of the three sections, clusters related to BP were discussed in depth. Remarkably, lipid and fat-related GO terms were not represented in the significant analysis of the GO enrichment of normal DEG. Hence, the sublist of genes was created to identify the lipid and fat-related BP terms that could have been profoundly affected by supplementation. The sublist genes of the CCD, CCL, and KP groups revealed that there were 821, 864, and 1,172 entries enriched significantly in the biological process category (p < 0.05) (Tables S1, S2, and S3), respectively. There was a significant association of biological process items with the lipid, sterol, and lipoprotein metabolic processes for each subset of genes shown in Figure 2.

The common GO terms observed between CCL versus CCD and CCD versus normal were the metabolic process of fat-soluble vitamin, neutral lipid, glycerophospholipid, and cellular lipid, glycerolipid biosynthetic process, lipid homeostasis, lipid catabolic process, and response to lipid. Similarly, the metabolic process of fat-soluble vitamin and neutral lipid, lipid localization, glycerolipid biosynthetic process, regulation of lipid biosynthetic process, cellular lipid catabolic process, and response to lipid were similar GO terms observed between KP versus CCD and CCD versus normal. Furthermore, the common GO terms between CCL and KP versus CCD were fat-soluble vitamin metabolic process, glycerolipid biosynthetic process, lipid transport regulation, and lipid response. The above-mentioned points indicate that KP can mimic the functional role of CCL, as most of the BP influenced by choline supplementation are also modulated by KP supplementation.

Fatty liver syndrome is a metabolic abnormality caused by the deficiency of methyl donors and biotin in the feed [21], which generally affects fast-growing broilers and caged layers [22]. In birds, the prime location of *de novo* lipogenesis (the conversion of glucose to triglycerides) is the liver that generates 80%–85% of total fatty acid synthesis, which is stored in the adipose tissue [23]. Thus, hepatic lipogenesis plays a direct role in lipid mobilization to the major triglyceride storage site, avian adipocytes [24]. This observation demonstrated that the fat mobilization and metabolism of birds largely depend on the liver function.

Fatty liver can be prevented by choline supplementation as it can mobilize the hepatic triacylglycerol in lipoproteins toward non-hepatic tissues [25], demonstrating the importance of dietary addition of CCL in the commercial diet. Previously, we had reported histopathological liver abnormalities in birds raised on a CCD diet, which were reversed by KP and CCL. Hence, the present trial was planned to delineate the gene expression pathways linked with the lipotropic effect of CCL and KP in broilers raised on a CCD diet by comparing thousands of mRNA expression levels obtained from liver samples using Agilent microarray. This microarray technology also provides a comprehensive analysis of the chicken liver gene expression profile with respect to lipid regulation, fatty acid oxidation, development-associated genes, cell membrane-associated genes, lipid metabolism, organ development, and fat mobilization.

The degree of lipid accrual in the liver depends on the balance between anabolism (*de novo* lipogenesis) and catabolism (beta-oxidation and lipolysis) of lipids. Based on the logarithmic fold change, some of the candidate genes (though non-significant) related to lipid metabolic processes were selected and presented in Table 1. We observed that the CCD diet upregulated the gene expression of lipase C (LIPC) in liver tissues compared to normal birds. On the contrary, liver tissues of birds supplemented with CCL and KP showed downregulation in the expression of the LIPC gene compared to the CCD diet group. However, the downregulation of LIPC fold change was better in KP than in CCL. The LIPC encodes the HL enzyme, which is found primarily in hepatocytes and endothelial cells of the liver. One of the principal functions of HL is to catalyze the hydrolysis of phospholipids and triglycerides of intermediate density (ID), low density (LD), and high-density (HD) lipoproteins [26–29]. Thus, it maintains the steady levels of ID, HD, and LD lipoproteins by regulating the level of triglycerides in the blood. It was also discovered to be a significant stimulant in *de novo* lipogenesis, as the upregulation and downregulation are associated with lipogenesis and reduced fatty liver, respectively [30]. Interestingly, some genes [ABCG5, ABCG8, acetyl-CoA carboxylase (ACACA), peroxisome proliferator-activated receptor gamma (PPARG), and ATP citrate lyase enzyme (ACLY)] involved in *de novo* hepatic lipogenesis were modulated by KP and CCL intervention. The CCD diet downregulated the ABCG5 and ABCG8 genes compared to the normal group. However, the above-mentioned genes were highly expressed in the KP group compared to the CCD diet and CCL groups. ABCG5 and ABCG8 are members of a subfamily (G Member 5 and 8) of ATP-binding cassette (ABC) transporters that move various molecules within the cellular membranes. Furthermore, in research studies, it was demonstrated that mice with mutant alleles (ABCG5 and ABCG8) showed an increase in PPARG expression, which plays a critical role in *de novo* lipogenesis [31,32]. PPARG, ACACA, and ACLY were downregulated in the KP and CCL-treated groups, whereas PPARG was upregulated in the CCD diet. Although the fold change of PPARG is similar in KP and CCL, the negative regulation of ACACA and ACLY was more prominent in KP when compared to CCL. PPARG is a member of the nuclear hormone receptor superfamily of ligand-activated transcription factors [33]. Triglyceride homeostasis is controlled in part by PPARG [27–34].

**Figure 3.**
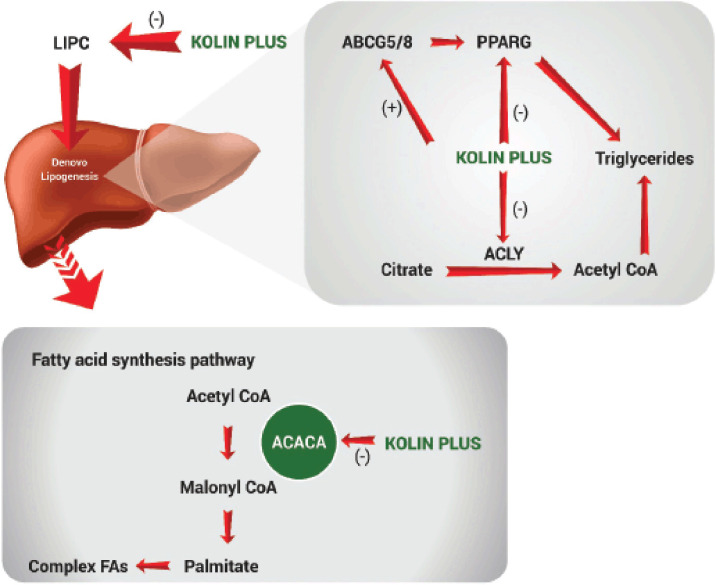
Effect of KP on gene expression related to *de novo* lipogenesis.

The metabolite acetyl-CoA participates in several important biosynthetic pathways, including fatty acid synthesis. It is synthesized by the cytosolic ACLY from citrate with concurrent hydrolysis of ATP to ADP and phosphate [29]. Similarly, ACACA, a complex multifunctional biotin-containing enzyme system and a rate-limiting enzyme in lipogenesis as it catalyzes the carboxylation of acetyl-CoA to malonyl-CoA [35]. Thus, KP regulated liver lipid accumulation by modulating the LIPC, ABCG5, ABCG8, ACACA, ACLY, and PPARG genes involved in *de novo* lipid synthesis (Fig. 3).

**Figure 4.**
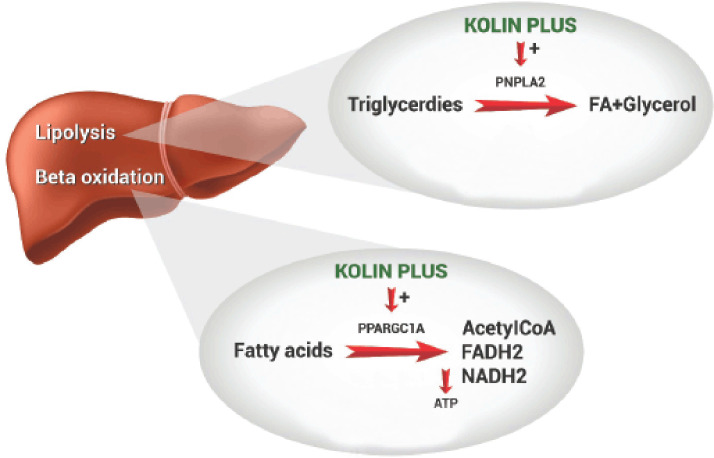
Effect of KP on catabolism of lipids (lipolysis and beta-oxidation of fatty acids).

**Figure 5.**
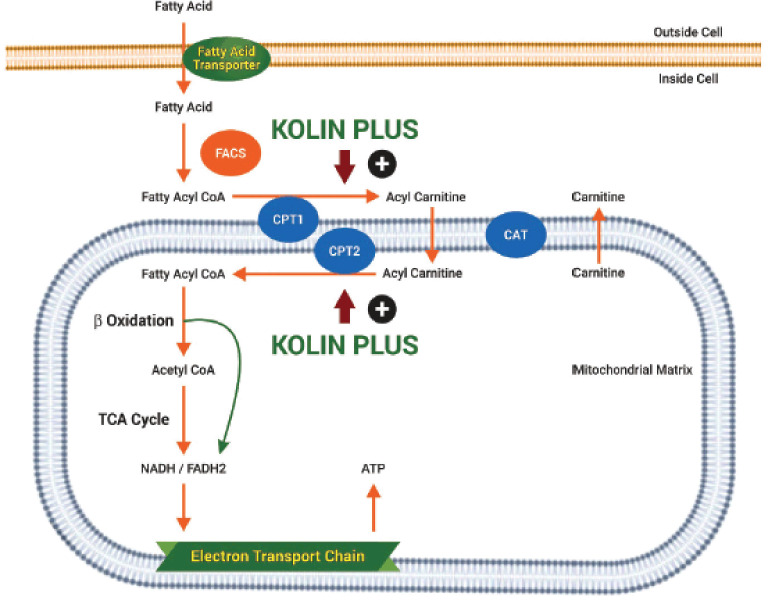
Effect of KP on genes related to beta-oxidation of fatty acids.

Furthermore, we identified the genes that play a critical role in the beta-oxidation of fatty acids and lipolysis pathways. Peroxisome proliferator-activated receptor gamma coactivator 1 alpha (PPARGC1A), carnitine palmitoyl transferase I alpha (CPT1A), patatin-like phospholipase domain containing 2 (PNPLA2), and hydroxyacyl-CoA dehydrogenase trifunctional multienzyme complex subunit beta (HADHB) genes were upregulated in KP and CCL, but only the PNPLA2 gene was downregulated in the CCD group. Moreover, the fold changes of PPARGC1A, CPT1A, PNPLA2, and HADHB were higher in KP than in the CCL-supplemented group. PPARGC1A is the master regulator of mitochondrial biogenesis and oxidative metabolism, lipogenesis, and triglyceride secretion. Morris et al. [36] stated that overexpression of hepatic PPARGC-1A results in increased fatty acid oxidation with reduced triacylglycerol storage and secretion in the liver. CPT1 (located in the outer membrane and is detergent-labile) is responsible for transporting long-chain fatty acids across the mitochondria for beta-oxidation-carnitine transport [37], and its deficiency may result in a decreased rate of fatty acid beta-oxidation. The HADHB gene encodes the beta subunit of the mitochondrial trifunctional protein (MTP), which catalyzes the last three steps of mitochondrial beta-oxidation of long-chain fatty acids. Nassir et al. [38] reported that fatty acid oxidation was decreased in MTP knockout mice compared to wild-type mice. Adipose triglyceride lipase (ATGL) is a major hepatic triacylglycerol lipase encoded by the PNPLA2gene, which catalyzes the first step in the hydrolysis of triglycerides and provides the signal to control energy metabolism. Recent studies have discovered that high PNPLA2 levels are required for efficient hepatic lipid metabolism, as the absence of hepatic ATGL causes steatosis [39,40]. The above-mentioned data substantiates that PPARGC1A, CPT1, HADHB, and PNPLA2 genes play a significant role in liver lipid metabolism, which is regulated by KP supplementation (Figs. 4 and 5).

**Table 1.** Representation of selected gene expression fold change.

Genes	Normal versus CCD (logarithmic fold change)	*p*-value	KP versus CCD (logarithmic fold change)	*p*-value	CCL versus CCD (logarithmic fold change)	*p*-value
LIPC	1.491	0.003	−1.577	0.004	−0.933	0.054
ABCG5	−1.315	0.236	1.217	0.045	−0.049	0.482
PPARG	0.566	0.115	−0.758	0.001	−0.722	0.004
ACLY	0.055	0.609	−1.013	0.026	−0.378	0.382
CPT1A	0.318	0.107	0.638	0.006	0.366	0.327
PPARG1A	0.067	0.603	1.297	0.079	0.755	0.273
PNPLA2	−0.393	0.296	0.843	0.050	0.372	0.127
HADHB	0.102	0.378	0.777	0.29	0.244	0.618
ABCG8	−0.875	0.244	0.970	0.120	−0.199	0.760
ACACA	0.364	0.480	−0.894	0.128	−0.630	0.068

Gene-fold change is provided in terms of logbase2 and is expressed as means of three replicates (2 *equimolar* pooled samples/replicate).

Taken together, the current results reveal that the KP might regulate lipid accumulation by controlling the expression of genes (LIPC, ABCG5, ABCG8, ACACA, ACLY, PPARG, PPARGC1A, CPT1, HADHB, and PNPLA2) involved in *de novo* lipogenesis and fatty acid oxidation in chicken liver tissues. Moreover, the impact of KP supplementation on the gene expression fold change was much better than that of the CCL-supplemented group. The phytoconstituents (polyphenols and curcuminoids) present in KP could be attributed to the modulation of the above genes responsible for lipid metabolism/catabolism and lipogenesis in the liver as the different ingredients in KP (*A. nilotica* and *C. longa)* have been already reported to have hepatoprotective activity [40–42]. So, the microarray analysis confirms that KP (better than CCL) supplementation changed the way the CCD diet changed transcriptional changes related to lipid regulation in the liver. This was also shown by chicken performance traits and fatty liver [19].

## Conclusion

The present results showed that dietary KP supplementation modulated the expression of genes responsible for fatty acid oxidation by regulation of PPARGC1A, CPT1, HADHB, and PNPLA2 genes and in lipogenesis through the regulation of LIPC, ABCG5, ABCG8, ACACA, ACLY, and PPARG genes in the liver.

## Data Availability

The microarray data were submitted in NCBI Gene Expression Omnibus (GSE193116).

## List of Abbreviations

Biological Process (BP), Gene ontology (GO), Hepatic lipase (HL), Adenosine diphosphate (ADP), Choline chloride-deficient (CCD), Choline chloride (CCL), Kolin Plus^TM^ (KP).

## Supplementary Material

S1 Link: https://docs.google.com/spreadsheets/d/1tVRuue06kOStpYHiGspsBiYLLpyAThwV/edit?usp=sharing&ouid=117212304002401654430&rtpof=true&sd=true

S2 Link: https://docs.google.com/spreadsheets/d/1y2bs-5snK_Oc-iHMlq3bp6pRUrD2euNp/edit?usp=sharing&ouid=117212304002401654430&rtpof=true&sd=true

S3 Link: https://docs.google.com/spreadsheets/d/1IJx8Pj4aN5b7IZYidX4ZZC96m_D9Gfrl/edit?usp=sharing&ouid=117212304002401654430&rtpof=true&sd=true
